# Construction and optimization of a genetic transformation system for efficient expression of human insulin-GFP fusion gene in flax

**DOI:** 10.1186/s40643-024-00799-9

**Published:** 2024-08-27

**Authors:** Wei Zhao, Rui Zhang, Luyang Zhou, Zhongxia Zhang, Fei Du, Ruoyu Wu, Jing Kong, Shengjun An

**Affiliations:** 1 School of Medicine, Hebei University of Engineering, Handan Economic and Technological Development Zone, No. 19 Taiji Road, Handan, Hebei Province 056038 China; 2 Hebei Provincial Engineering Laboratory of Plant Bioreactor Preparation Technology, Hebei University of Chinese Medicine, No. 326 Xinshi South Road, Qiaoxi District, Shijiazhuang, Hebei 050090 China; 3 The Second Hospital of Hebei Medical University, No. 215 Heping West Road, Changan District, Shijiazhuang, Hebei 050000 China; 4 Shijiazhuang Medical College, No.1 Tongxin Road, Lingshou County, Shijiazhuang, Hebei 050500 China; 5 Department of Ultrasound Medicine, Hengshui People’s Hospital, Hengshui, Hebei 053000 China

**Keywords:** Flax callus, Insulin-GFP, Genetic transformation, *Agrobacterium*-induced infection, Protoplasts

## Abstract

The human *insulin gene* modified with a C-peptide was synthesized according to the plant-preferred codon, and a *fusion gene* expression vector of insulin combined with green fluorescent protein (GFP) was constructed. The optimization of the flax callus culturing was undertaken, and a more efficient *Agrobacterium*-mediated genetic transformation of the flax hypocotyls was achieved. The critical concentration values of hygromycin on the flax hypocotyl development, as well as on its differentiated callus, were explored by the method of antibiotic gradient addition, and the application of antibiotic screening for the verification of positive calluses was assessed. The *fusion gene* of insulin and GFP was successfully inserted into the flax genome and expressed, as confirmed through polymerase chain reaction and Western blotting. In conclusion, we have established a flax callus culture system suitable for insulin expression. By optimizing the conditions of the flax callus induction, transformation, screening, and verification of a transgenic callus, we have provided an effective way to obtain insulin. Moreover, the herein-employed flax callus culture system could provide a feasible, cheap, and environmentally friendly platform for producing bioactive proteins.

**Graphical Abstract:**

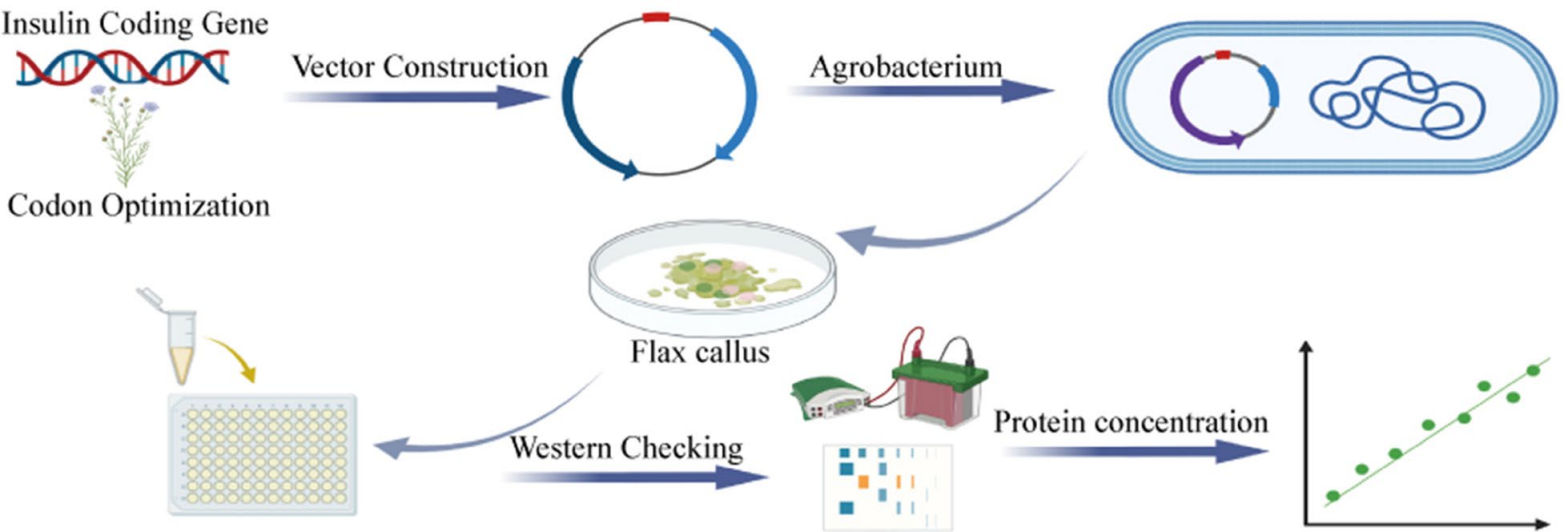

**Supplementary Information:**

The online version contains supplementary material available at 10.1186/s40643-024-00799-9.

## Introduction

Flax is an annual herb with a long history of cultivation (Lawrence et al. 2010). Its plant fibers can be used in fabrics, and its linseed oil can be used in food and medicine (Cummins 2004; Flower et al. 2014). Flax is a highly self-pollinating crop, effectively avoiding the potential risk of pollen drift (Yao et al. 2015; Sudarshan et al. 2017). The flax genome, known for its compact size and amenability to genetic manipulation (Zhan et al. 1988; Emets et al. 2009), has undergone progressive elucidation through sequencing efforts. Notably, the genomic sequence of the CDC Bethune cultivar of oilseed flax was first successfully deciphered in 2012, as reported by Kumar (Kumar et al. 2012) and independently confirmed or augmented by Wang (Wang et al. 2012). Recently, the Gansu Academy of Agricultural Sciences has sequenced several different varieties of flax to obtain chromosome-level reference genomes. The results of this genome sequencing are more conducive to applying genetic engineering technology in the genetic transformation of flax (Zhang et al. 2020); this is an excellent advantage for the rapid and mass production of target products. Many researchers have studied the tissue culture conditions and the genetic transformation related to flax (McHughen and Swartz 1984; Obert et al. 2009; Kesiraju et al. 2020). The research results of the method provide a foundation for us, allowing us to use flax as an exogenous protein expression platform (Yadav et al. 2022). Based on previous experience, our research group has explored the conditions of flax tissue culture and the genetic transformation methods according to the flax varieties used for research purposes. Preliminary studies have shown that the best medium for the callus induction and the subculture expansion of the hypocotyls of Longya-10 consisted of Murashige and Skoog Medium (MS) combined with 0.1 mg/L 1-Naphthaleneacetic acid (NAA) and 2 mg/L 6-Benzylaminopurine (6-BA), while the subculture period was 2–3 weeks. The critical concentration of antibiotic resistance in the callus was determined by regularly weighing the proliferation of the callus. It is advisable to coculture for 3 days after the *Agrobacterium* infection, and cefotaxime sodium is suitable for bacteriostatic antibiotics. These experimental results laid a solid foundation for culturing the flax callus and its use for insulin transformation and expression.

Insulin is a protein hormone secreted by pancreatic beta cells, consisting of 51 amino acids. It is divided into two polypeptide chains (A and B) linked through disulfide bonds (Khosravi et al. 2022). The primary role of insulin is to regulate glucose metabolism for the treatment of diabetes, which is a chronic disease (O’Mara et al. 2020). According to the International Diabetes Federation (IDF), the global prevalence of diabetes is estimated to be 10.5% (affecting 536.6 million people) in 2021, and it is expected to rise to 12.2% (thereby affecting 783.2 million people) by 2045. Meanwhile, global diabetes-related health spending is estimated at $966 billion in 2021 and is projected to reach $1,054 billion by 2045 (Sun et al. 2022). The use of insulin lies at the heart of diabetes treatment, and its role cannot be ignored. The blood glucose levels of patients with type-1 diabetes, gestational diabetes, and type-2 diabetes are still poorly controlled after acute complications, severe chronic complications, surgical infection, other stress states, and after multidrug combination therapy (Rasic-Milutinovic et al. 2008; Haque et al. 2020). Insulin therapy should be initiated when blood sugar is high in patients with newly diagnosed type-2 diabetes mellitus (Ghosal et al. 2017). The insulin used for the clinical treatment of diabetes is mainly biosynthetic human insulin or its analogs (Aggarwal 2011).

Due to the advantages of low cost, the feasibility of large-scale production, the lack of risk of contamination by mammalian pathogens, and the ability to post-translationally modify expressed proteins, plants are gradually becoming a crucial recombinant protein production platform (Gerszberg et al. 2022; Schillberg et al. 2022; Kowalczyk et al. 2022). Transgenic plants are increasingly used as natural sources for recombinant protein production (Vitale and Pedrazzini 2005). Bioreactors are also getting more and more attention. The development of plant transgenic technology provides a new opportunity for the biosynthesis of medicinal proteins. Various medicinal proteins have been transgenic and successfully used in the clinic (Yoshida and Shinmyo 2000; Kowalczyk et al. 2022).

Currently, platforms for plant insulin expression include Arabidopsis seeds (Nykiforuk et al. 2006), potatoes (Arakawa et al. 1998), and tobacco and lettuce leaves (Boyhan et al. 2011). Unlike previous plant expression systems, the advantages of using flax callus as a plant bioreactor for insulin expression are: (1) the flax callus culture system has several benefits, including rapid growth, the use of a controlled bioreactor to achieve protein consistency in a closed environment, fewer pathogen contamination problems involving viral or bacterial toxins, and the ability to address regulatory and environmental concerns related to the potential release of transgenic plants. In addition, extracting and purifying proteins from cell cultures is more straightforward, convenient, and cost-effective than extracting and purifying proteins from whole plants. (2) the flax callus system can be easily transformed into a suspended cell with oral potential. This study used flax callus culture technology and the Agrobacterium-mediated genetic transformation method to obtain positive flax callus containing the target protein and explore the technique of producing insulin in flax. Positive flax callus was identified using PCR, Western blot techniques, and GFP fluorescence observation. After obtaining positive flax callus tissue, continuous screening and subculture were carried out to obtain stable positive flax callus expressing insulin. At the same time, we completed the supervision culture of insulin expression, subcellular localization, and pure line cell cluster screening in flax callus tissue. This study explores for the first time the method of stable expression of insulin in flax callus tissue.

## Materials and methods

### Vector construction

The human *insulin gene* modified with C-peptide was synthesized according to the plant-preferred codons, where the insulin B-chain was connected to the A-chain through a propyl-propyl-lysine tripeptide (international patent: WO2004/111244). The barley cysteine protease signal peptide (Sun et al. 2011) and the human *insulin fusion gene* sequence were synthesized on the plant binary expression vector pSuper1300-green fluorescent protein (GFP) by GENEWIZ (China). They were driven by the super promoter (Huang et al. 2018). Firstly, the intermediate vector pUC57 (where the *fusion gene* was located) was double digested with *Xbal* and *Kpnl* to obtain a *fusion gene* fragment with restriction sites containing sticky ends at both ends. The pSuper1300-GFP plasmid was also double digested with *Xbal* and *Kpnl*. The digested product was purified and recovered by 1% agarose gel electrophoresis, and subsequently, a T4 DNA ligase was added to carry out the ligation reaction. The ligation was performed at 16 °C overnight. The ligation product was transformed into DH5α *Escherichia coli* competent cells and was evenly spread on an LB solid medium containing 50 µg/mL kanamycin. Through antibiotic screening, the single colony that grew was picked for polymerase chain reaction (PCR) identification, and the correct bacterial solution was identified and sent to the laboratory for sequencing. The sequenced correct recombinant plasmid was named “BIG.” Grown colonies were detected through PCR by using the specific forward and reverse primers (Insulin F: 5′- GCACTCTACTTGGTGTGTGGTG − 3′; Insulin R: 5′- GGTGCTCAGGTAGTGGTTGTC − 3′).

2.2 Induction and proliferation of flax hypocotyl callus.

According to Salaj J’s culture method (Salaj et al. 2005) and the previous experience of our research group, the technique of using flax hypocotyl to induce callus was as follows. We picked out the flax seeds and placed them in 75% ethanol for 1 min; we shook the solution gently to make sure the disinfectant entirely covered the surface of the seeds, then washed with sterile water 3 times for 2 min each time and then put the seeds in sodium hypochlorite with an effective chlorine content of 5%. After the seeds were sterilized twice (for 2 min each time), they were washed with sterilized water 6−8 times. The seeds were then spread on an MS solid medium and cultivated in the dark, at 25 °C, until germination. Once the hypocotyl grew to 6–8 cm, we cut it into 0.5 to 1 cm-long pieces and placed the small pieces into the callus induction medium (MS supplemented with 0.1 mg/L NAA and 2 mg/L 6-BA) for about 2 weeks. Consequently, the hypocotyl formed a callus and was sub-cultured every 2–3 weeks. The unaltered (normal) induced flax callus was used as a negative control.

### Assessment of the sensitivity to antibiotics in the callus induction and proliferation stages of the non-transgenic hypocotyl differentiation

#### Callus induction stage of non-transgenic hypocotyl differentiation

Hygromycin-containing hypocotyl-induced callus medium was prepared with the addition of 0, 10, 20, 30, 40, 50, 60, 70, 80, 90, 100, 110, 120, and 130 mg/L of hygromycin. Bottles were inoculated with 10 hypocotyls, respectively, and 3 bottles were treated with each concentration; therefore, 42 bottles were used. The callus induction was observed after 25 days, and the critical concentration of hygromycin in the induction stage of flax hypocotyl was determined.

#### Proliferation stage of the non-transgenic callus

Hygromycin-containing hypocotyl-induced vigorously proliferating callus medium was prepared with the addition of 0, 10, 20, 30, 40, 50, 60, 70, 80, 90, and 100 mg/L of hygromycin. Each concentration was applied to 3 bottles; therefore, 33 bottles were used. Each bottle was inoculated with 1 g (fresh weight) of vigorously growing callus, and after 25 days, the fresh weight increase of each bottle was measured. The same group of treatments with no fresh weight increased and decreased was selected to study the critical hygromycin resistance in the callus proliferation stage value.

### Establishment and optimization of the flax genetic system mediated by *Agrobacterium tumefaciens*

#### *Agrobacterium*-induced infection of the flax hypocotyls

The BIG plasmid was transformed into *Agrobacterium* GV3101 competent cells through the liquid nitrogen freeze-thaw method (Wu et al. 2015).

#### Genetic transformation of the flax hypocotyls

The flax Longya-10 was selected. Flax seeds were sterilized and plated on MS medium until they germinated. Once the hypocotyls grew between 6 and 8 cm, they were cut into 0.5 to 1 cm-long pieces. We centrifuged the activated *Agrobacterium tumefaciens* GV3101 (with an OD_600_ value of 0.6–0.8) at 5,000 rpm for 5 min. After discarding the supernatant, the cell pellet was resuspended with an equal volume of MS liquid medium, and then, the flax hypocotyls cut into small pieces were soaked in the resuspension solution and shaken for 20 min. The hypocotyls were removed from the infection solution and placed on a sterile filter paper to dry. The hypocotyls were placed on a coculture medium (MS supplemented with 0.1 mg/L NAA and 2 mg/L 6-BA) for 3 days in the dark. Subsequently, the hypocotyls were transferred to a callus induction medium containing 80 mg/L hygromycin and 500 mg/L cefotaxime sodium. About 2 weeks later, resistant embryogenic calluses began to appear on the hypocotyls. During this process, the growth of the hypocotyls was observed, and the hypocotyls of the bacteria were removed in time. The resistant calluses in the differentiation process were subcultured once every 2 weeks, the browned tissue pieces were continuously removed, and the freshly grown calluses were retained for sub-culturing. The dosage was adjusted until the bacteriostatic antibiotics were removed entirely. Screening antibiotics were continuously added to the medium to ensure adequate screening pressure.

#### Fluorescence observation of resistant calluses

The transformed explants were observed by a LUYOR-3415RG (Luyang Instrument Co., Ltd, China) excitation light source; the fluorescence was monitored, and the expression of the target protein was assessed.

#### PCR- and RT-PCR-mediated identification of resistant calluses

When the calluses screened by antibiotics entered the proliferation stage, we selected the fresh and vigorously proliferating resistant calluses, used the plant genome DNA extraction kit (Tiangen, China) to extract their genome, and used the FastHiFi PCR MsdterMix (TAKARA, Japan) to carry out PCRs. After extracting the RNA with the plant RNA extraction kit (Tiangen, China) based on the FastKing gDNA Dispelling RT SuperMix (Tiangen, China) instructions, the extracted wild-type/positive flax callus RNA was reverse transcribed. The PCR was performed after the cDNA was obtained. Electrophoresis was performed on a 1% agarose gel. The primers for detecting the *insulin gene* were the same as before, and the length of the target gene was 735bp.

#### Western blotting for the detection of resistant calluses

Select the callus that can emit green fluorescence under an excitation light source, place it in a mortar, add liquid nitrogen, and grind it into powder with a pestle. The powder was added to plant RIPA lysate containing protease inhibitor (Beyotime, China), mixed by shock, and cracked on ice for 10 min. The mixture was then centrifuged at 12,000 rpm for 10 min at 4 °C. Took supernatant, mixed with protein loading buffer, and then boiled at 100 °C for 5 min. Let it cool to room temperature, and then screen through Western blotting. Tris-Tricine-SDS-PAGE gel with separation glue of 16.5% and concentration glue of 4% was used for electrophoresis. After electrophoresis, the polyvinylidene fluoride (PVDF) membrane was used to transfer the membrane using a wet membrane transfer apparatus. The membrane was then incubated with a 5% skimmed milk powder solution at room temperature for 2 h as a blocking treatment. We subsequently added a mouse antihuman insulin primary antibody (ThemoFisher, USA) that was diluted (1:100) in Tris-HCl buffer solution + Tween20 (TBST) and incubated overnight at 4 °C. On the next day, the PVDF membrane was taken out, the membrane was washed three times with TBST for 10 min each time, and an anti-mouse secondary antibody Goat Anti-Mouse IgG (H + L) (TransGen, China) diluted (1:5,000) in TBST was added, shook slowly at room temperature for an hour. The secondary antibodies were cleaned with TBST, cleaned three times for 10 min each time, then incubated with ECL luminescent solution for 4 min, and then entered the multifunctional imaging system (Fusion FX5 Spectra, France) for chemiluminescence.

#### Liquid chromatography with tandem mass spectrometry (LC-MS/MS) for the identification of proteins

The positive callus whole protein was extracted using the abovementioned method and subjected to SDS-PAGE electrophoresis. After electrophoresis, Coomassie brilliant blue was used for staining. The gel pieces were cut out at the corresponding positions and were sent to Qingdao Sci-tech Innovation Quality Testing Co. Ltd. for LC-MS/MS-mediated protein identification.

#### Preparation of the flax cell protoplasts and subcellular localization of the target protein

Protoplasts were prepared from loose positive calluses. The positive calluses were crushed in the ultraclean workbench, and 5 mL of the enzymolysis solution I (containing 1.5% cellulase R10, 0.75% macerozyme R10, 0.6 M mannitol, and 10 mM MES; pH 5.8) and 2 mL of enzymolysis solution II (containing 1.5% cellulase R10, 0.4% macerozyme R10, 0.4 M mannitol, and 10 mM MES; pH 5.8) were mixed with the flax calluses and vacuum filtered for 15 min. Enzyme hydrolysis was conducted in a dark environment for 7–8 h on a shaker with a rotation speed of 50 rpm at 30 °C. The protoplasts were filtered with a 40 μm filter, were placed into a centrifuge tube, and were centrifuged at 800 rpm, at 15 °C, for 4 min. At that point, a small amount of precipitation could be seen at the bottom of the centrifuge tube. After aspirating the supernatant, we washed the pellet twice with 1 mL of precooled solution I (containing 154 mM NaCl, 125 mM CaCl_2_, 2 mM KCl, 2 mM MES, and 5 mM glucose; pH 5.8) and centrifuged at 600 rpm for 4 min, at 15 °C. We then carefully aspirated the supernatant, gently shook the precipitation with 1 mL of precooled solution I, and let it stand for 30 min before centrifuging it again at 600 rpm, 4 min, at 15 °C. We then carefully aspirated the supernatant again and took an appropriate amount of solution III (containing 0.4 M mannitol, 15 mM MgCl_2_.6H_2_O, and 4 mM MES; pH 5.8) to suspend the flax protoplasts. By using 100 µL of the above-obtained protoplasts, we added 10 µL of the endoplasmic reticulum marker plasmid, took a solution II (containing 0.4 M mannitol, 100 mM CaCl_2_, and 40% PEG4000; pH 5.8) equal to the sum of the two volumes, mixed gently and placed them in a water bath set at 30 °C, for 15 min. The endoplasmic reticulum localization signal protein used was Sper, and its amino acid sequence was MKTNLFLFLFLIFSLLLSLSSAEF. Subsequently, 1 mL of solution I was mixed with the protoplast solution to terminate the reaction. Protoplasts were collected by centrifugation at 600 rpm for 4 min; the supernatant was carefully removed, and 1 mL of solution I was added to wash the protoplasts 1−2 times. Finally, 1 mL of solution I was added to the solution, and after culturing in the dark at 30 °C for 18−24 h, we centrifuged the protoplast solution at 600 rpm for 4 min. The supernatant was then aspirated, leaving only 100 µL of protoplasts for observation through laser confocal microscopy.

#### Single-cell and cell-cluster clone screening

The nursing culture method used in this study is according to the previous reports(Chabane et al. 2007). A piece of actively growing flax callus (the “nursing callus”) was placed on the medium. Subsequently, a circular sterile filter paper with a diameter of about 1 cm was placed on the nursing callus. A filter paper with a smaller diameter allowed the cells to be cultured and make the most of these secretions. The nursing callus with the filter paper was pre-cultured for 2 days so that the filter paper fully absorbed and diffused the callus secretions. The loose positive callus was crushed lightly and cultured in liquid medium (containing MS supplemented with 0.1 mg/L NAA and 2 mg/L 6-BA) on a shaker set at 120 rpm, in the dark, for 6–8 days. Pipetted single cells or small cell clusters in the culture medium and inoculated them on filter paper for culture. When the cell clusters with a diameter of about 5 mm grow, they can be transferred to a solid medium for culture.

## Results

### Identification of the recombinant plasmid

**Fig. 1:**
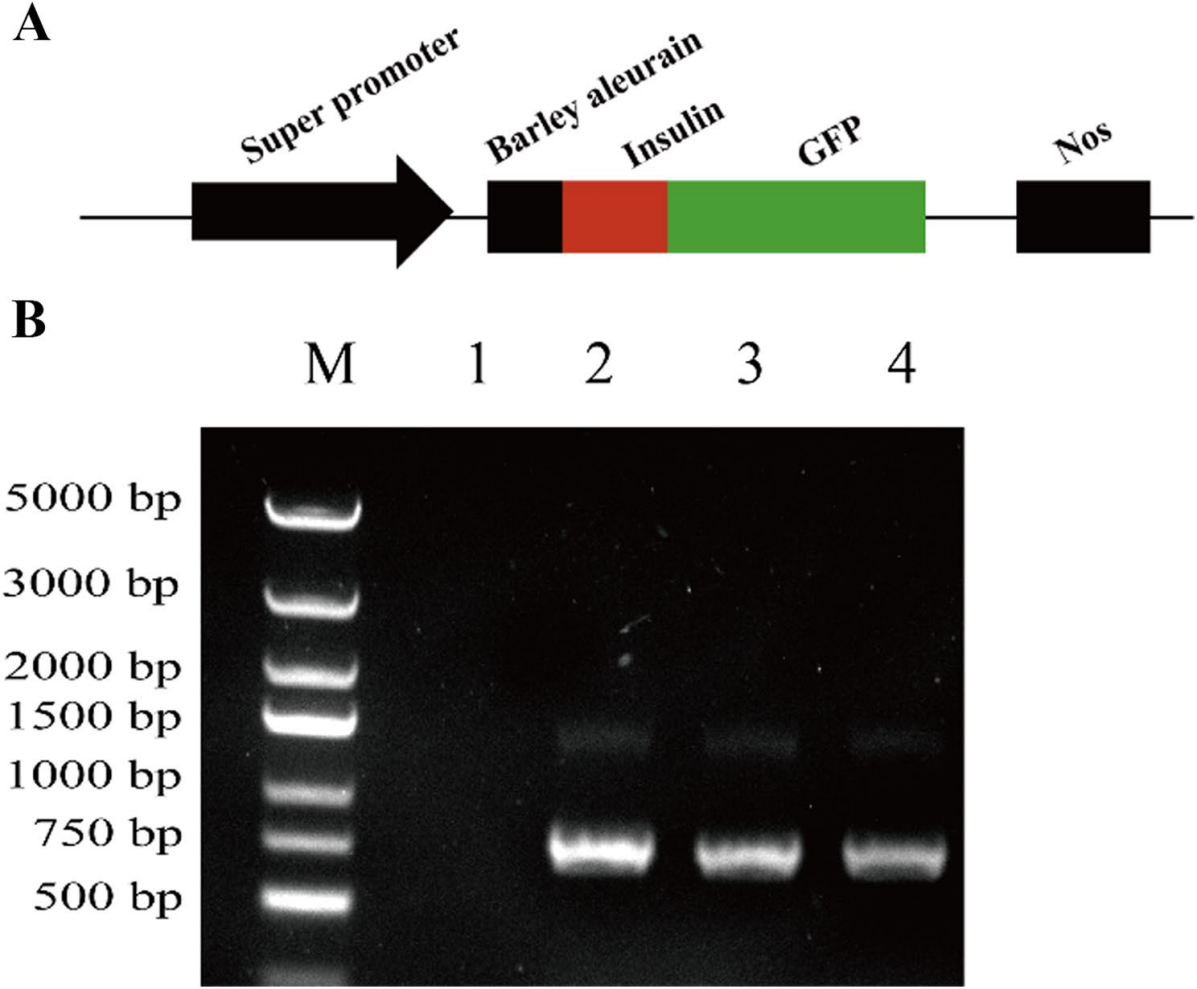
Identification of the recombinant plasmid Insulin-BIG. (**A**) Structural diagram of the recombinant plasmid BIG. (**B**) Enzyme digestion of recombinant plasmid and PCR-mediated verification of the transformant. M, DNA marker; 1, blank control; 2−4, recombinant plasmid BIG

The recombinant plasmid harbors a super promoter that can efficiently initiate the transcription. A barley cysteine protease signal peptide was added upstream of the *insulin gene* sequence to guide the transfer of the fusion protein to endoplasmic reticulum synthesis. The GFP sequence downstream of the *insulin gene* sequences could detect the expression of the fusion protein and mark positive tissues and cells. According to the central rule and the biosynthesis direction of the protein from the N-terminus to the C-terminus, detecting the GFP fluorescence can indicate the successful synthesis of the fusion protein containing insulin. A schematic of the structure of the plasmid BIG (Barley cysteine protease signal peptide, Insulin, GFP) is provided in Fig. 1A. The constructed plasmid BIG was identified by PCR, and the results are shown in Fig. 1B. The regions covered by the two primers included the BIG insulin, and the length of the *target gene* was 228 bp. The subsequent DNA sequencing results further confirmed that the recombinant plasmid BIG was successfully constructed and that the next step of the flax transformation experiment could be carried out.

### Flax plant chassis stable callus cultures and optimization of the flax genetic system mediated by *Agrobacterium tumefaciens* were established

**Fig. 2:**
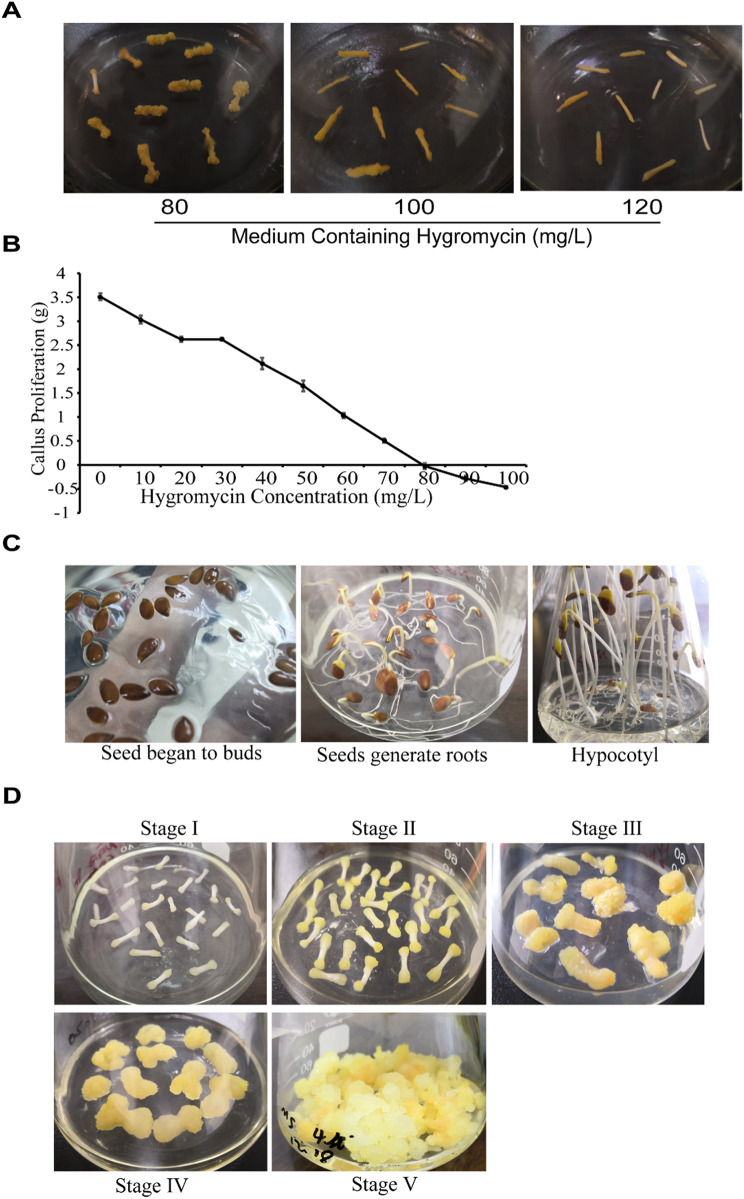
Callus formation in flax. (**A**) Effect of hygroscopic linseed on callus induction. The picture shows the growth of a flax hypocotyl on a callus induction medium containing hygromycin after 25 days, which exhibited a characteristic state in different concentrations of hygromycin as indicated. (**B**)Effect of hygromycin on the callus proliferation of flax. The curve shows the change value of the callus proliferation with the increase of hygromycin concentration in the culture medium. When the hygromycin concentration was 80 mg/L, the callus proliferation became negative. (**C**) Callus formation process in flax: the seeds begin to bud; the seeds generate roots and sprouts; hypocotyl elongation to 6–8 cm. (**D**) Describe the five stages for hypocotyl induction: both ends of the hypocotyl segments begin to expand (stage I); an obvious swelling and bulge forms at both ends of the hypocotyl segments (stage II); a complete enlargement of the hypocotyl segments occurs (stage III); callus formation takes place (stage IV); calluses with exuberant growth are identified after three subcultures (stage V)

The growth state of hypocotyls in the medium containing various concentration gradients of hygromycin gradually deteriorated as the concentration increased. With the increase of the concentration of hygromycin in the medium, the inhibitory effect was gradually apparent, the time for the emergence of the callus was delayed, and the proliferation of the callus tissue slowed down. When the concentration reached a specific value, hygromycin exhibited an apparent inhibitory effect on the induction of the hypocotyl callus of flax. However, when the concentration reached 80 mg/L, all the calluses that healed were yellow-brown, shrunken on the surface, withered in shape, and stagnant in growth. When the concentration reached 100 mg/L, the hypocotyls began to fail to heal. Finally, when the concentration increased to 120 mg/L, the healing rate of the hypocotyl was close to zero (Fig. 2A). Therefore, 120 mg/L of hygromycin was considered the critical concentration value for the flax hypocotyls to induce calluses. In general, the inducing critical concentration should be used as the resistance screening concentration for screening resistant calluses; however, when the vector was marked with GFP, the screening of the positive calluses would become more accurate. Moreover, the antibiotics adding a selection pressure that was too high would also adversely affect the subsequent proliferation of the positive callus that emerged and even reduce the positive transformation rate. Therefore, this experiment adopted the concentration of 80 mg/L of hygromycin as a screening concentration at the initial stage of the hypocotyl induction.

As shown in Fig. 2B, adding a certain amount of hygromycin significantly inhibited the proliferation of the flax calluses. As the concentration of hygromycin increased, the proliferation of the flax callus growth decreased accordingly. When the hygromycin concentration reached 80 mg/L, the flax callused exhibited a negative growth phenomenon; that is, the fresh weight of the flax callus after 25 days decreased by an average of 0.03 g when compared with the fresh weight of the initial inoculation. Therefore, when screening positive flax calluses, a concentration of 80 mg/L of hygromycin should be the screening concentration for flax callus resistance.

We planted the sterile flax seedlings (Fig. 2C), removed the hypocotyls, cut them into 0.5 to 1 cm-long pieces, and placed them on the callus induction medium for cultivation. After approximately 5 days, the hypocotyls swelled and healed in about 15 days. Wound tissue formed and continued to grow, and after 2 passages, vigorously proliferated calluses were formed (Fig. 2D).

### Flax cells could play high-efficiency chassis roles to express functional foreign protein

**Fig. 3:**
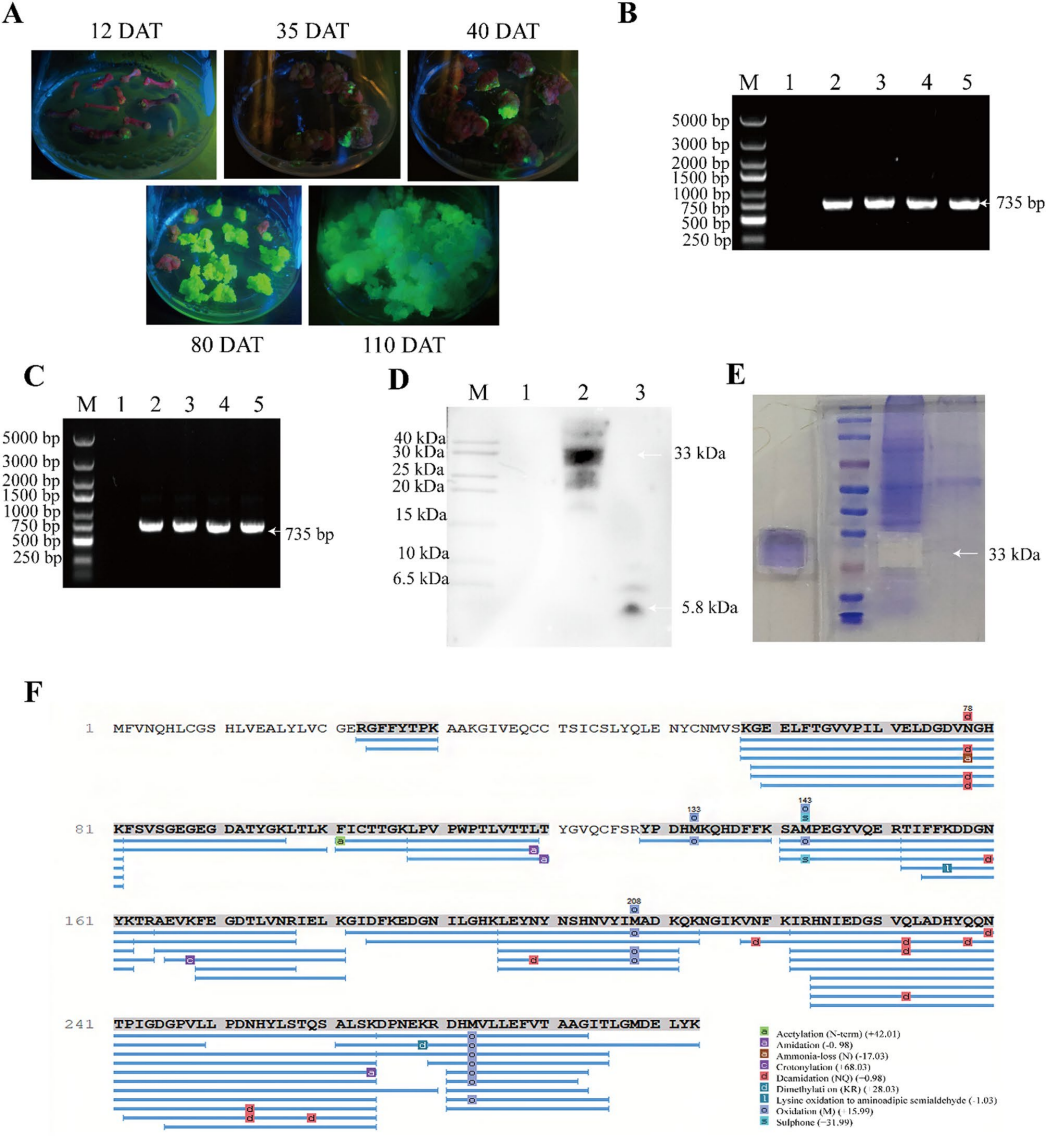
Flax cells as effective plant chassis for expression of the target protein. (**A**) Generation of positive calluses and fluorescence screening subculture under a LUYOR-3415RG fluorescent light source. Resistant calluses begin to form 12, 35, 40, 80, and 110 days after transformation (DAT), respectively. The fluorescent calluses had the advantage of proliferation, and their proliferation was found to be increasing. (**B**) PCR analysis of positive calluses. Gene levels’ detection in the third-generation calluses with positive GFP fluorescence. The selection of three pieces of DNA for PCR identification. M, DNA marker; 1, negative control of the wild-type flax callus; 2–4, resistant callus DNA; 5, positive amplification control. (**C**) RNA was extracted from three resistant calluses and reverse transcribed for PCR verification. M, DNA marker; 1, negative control of the wild-type flax callus; 2–4, resistant callus cDNA; 5, positive control of the amplification. (**D**) Western blotting analysis of positive calluses. Protein level detection of the third-generation calluses with positive GFP fluorescence detection. The total protein of the calluses was extracted and analyzed by Western blotting using a primary insulin antibody. M, protein marker; 1, wild-type flax callus; 2, resistant callus; 3, insulin as positive control. (**E**) This is the result of Coomassie Blue staining. LC-MS/MS-mediated identification of the fusion protein. A gelatin block for detecting the fusion protein (Western blotting results confirmed) at a molecular weight of 33 kDa. (**F**) The map for the identification of the fusion protein by LC-MS/MS. The blue line shows the corresponding peptide segments and times detected by mass spectrometry, while the colored letters indicate the protein modification sites

When the hypocotyl was 6–8 cm long, we cut it into 0.5 to 1 cm-long pieces. After being infected with *Agrobacterium*, the hypocotyl was cultured on the callus induction medium. After approximately 2 weeks, some hypocotyls were found to grow calluses. LUYOR-3415RG was used to stimulate the light source to observe the protein expression. After picking out the infected hypocotyls, we continued to observe the growth. It was found that some hypocotyls would turn brown and wither during the induction process, while some other hypocotyls were only partially able to form calluses (Fig. 3A).

The transformed explants were observed with a LUYOR-3415RG excitation light source, and their fluorescence was monitored. The fusion protein was expressed during the callus induction and the vigorous proliferation stages (Fig. 3A).

### Flax cell chassis successfully express human insulin

The callus genomic DNA was extracted and tested through PCR. The length of the *target gene* (BIG) was 735 bp. The agarose gel results are presented in Fig. 3B. Callus RNA was extracted and reverse-transcribed to obtain cDNA. PCR detection was performed with the same primers, and the agarose gel results are shown in Fig. 3C.

To verify the positive results further, a Western blot was carried out. In Fig. 3D, the loading amount of the total callus protein was 20 µL. The molecular weight of the insulin and GFP fusion protein was about 33 kDa, and a band appeared at the corresponding position. No bands appeared in the non-transgenic samples.

To determine the expression level of the target protein, 3 µL of protease inhibitor (100x) (Epizyme, China) was added to 300 µL of plant RIPA lysate (Beyotime, China), thoroughly mixed and pre-cooled on ice. A fluorescent callus weighing 100 mg was ground into a powder with liquid nitrogen. The supernatant of approximately 300 µL was obtained by vigorously mixing the lysate, incubating on ice for 10 min, and centrifuging at 12,000 rpm for 10 min at 4 °C. An appropriate amount was then diluted with sample diluent (Elabscience, China) by a factor of ten and set aside. An ELISA kit (Elabscience, China) was utilized with standard concentrations set at 50 µIU/mL,25 µIU/mL,12.5 µIU/mL, 6.25 µIU/mL, 3.13 µIU/mL, 1.57 µIU/mL, 0 0.78 µIU/mL. 0 µIU/mL (blank hole), with three sets of replicates provided for each standard concentration. The sample wells were also provided in triplicate. Additionally, 100 µL samples or standard products were added to each well and incubated at 37 °C for 90 min. The liquid in the wells was then discarded, and 100 µL biotin antibody-antigen working solution was added, followed by an additional incubation period at 37 °C for 60 min. Afterwards, the wells were washed three times. Enzyme binding working solution (100 µL) was added and incubated at 37 °C for 30 min, followed by five washes. Substrate solution (90 µL) was added and incubated at 37 °C for 15 min. Finally, a termination solution (50 µL) was added immediately before measuring OD values using a multi-function microplate reader (Varioskan LUX, China) at a wavelength of 450 nm. By analyzing the measured data and substituting the sample value into the standard curve, the sample concentration is determined to be 1172.7 mIU/g.

After the SDS-PAGE electrophoresis, we cut the gel block at the molecular weight position of the fusion protein (corresponding to the one identified in the Western blot; Fig. 3E) and sent it to Qingdao Kechuang Quality Inspection Co., Ltd. for LC-MS/MS-mediated protein identification. The corresponding molecular weight position was annotated on the gel block. The types of mixed protein were significantly reduced compared to the total protein of the flax callus. This improved the detection efficiency of the target protein. The protein in the gel block was hydrolyzed by trypsin and was cleaved into several peptide fragments at amino acids K and R. The mixed peptides were scanned by mass spectrometry, and the scan data was compared with the theoretical amino acid sequence of the fusion protein. The results revealed that 40 specific peptides covered 81% of the fusion protein. The matching results of the corresponding maps of the peptide fragments were good, and the protein confidence was high (Fig. 3F). As the detectability of peptides in mass spectrometry can be affected by several uncontrollable factors (Inglese et al. 2022), the undetected part may limit the detection accuracy. Therefore, subsequent experiments must be conducted to assess the purification method, and the purified protein can also be used to determine the amino acid sequence for the protein.

**Fig. 4:**
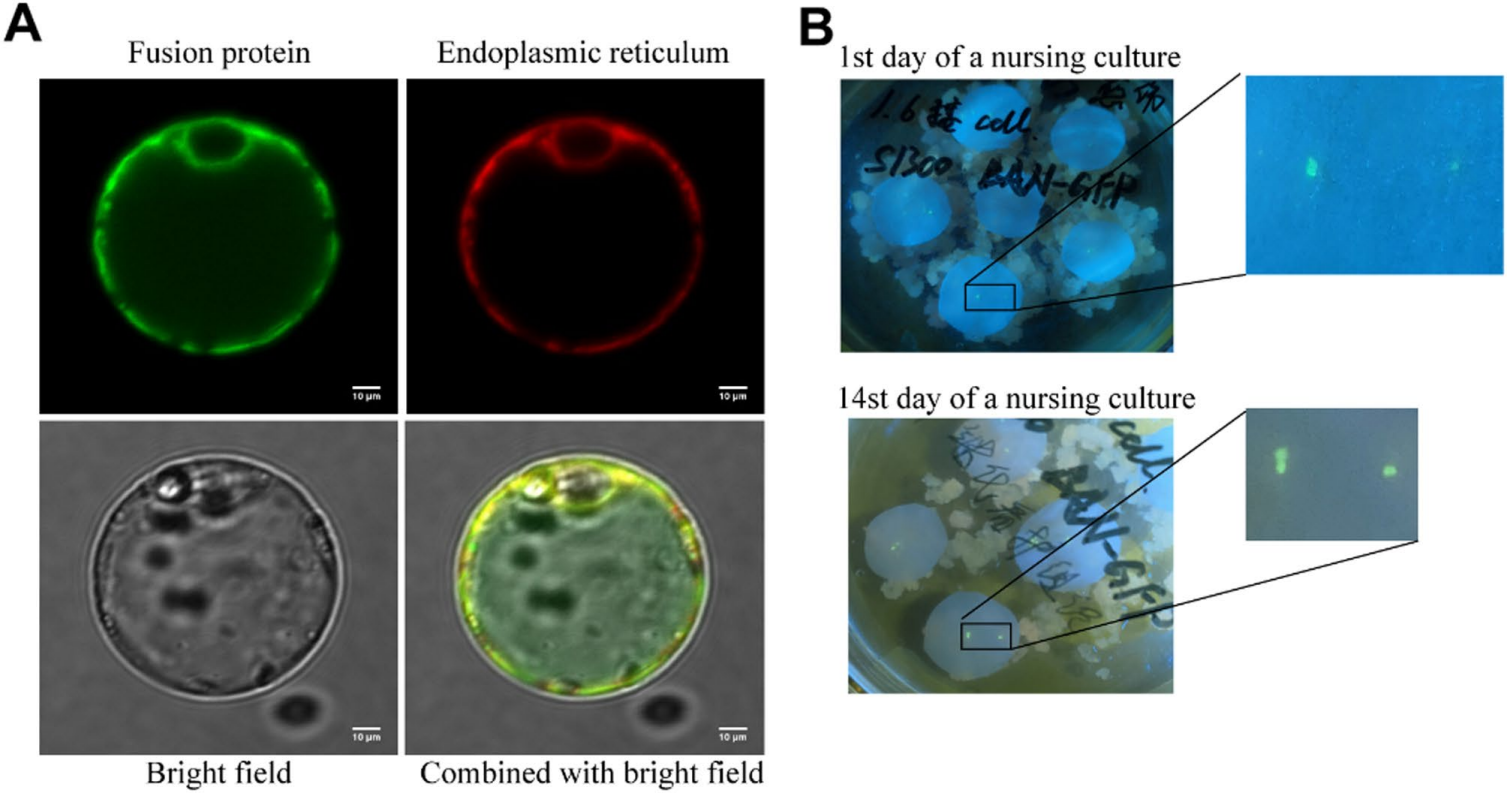
The detection of insulin-GFP protein. (**A**) The positive callus is prepared into protoplasts, and the aggregation position of the fusion protein is observed under a laser confocal microscope. (**B**) Nursing culture of a cell mass. Small cell clusters of a nursing culture’s 1st and 14th days were selected from the culture medium

Protoplasts were prepared from loose positive calluses. The protein expression was observed by a laser confocal microscope, as shown in Fig. 4A. The GFP was used to trace the fusion protein inside the endoplasmic reticulum.

To harvest more target proteins of uniform quality and generate cell lines with fast reproduction, stable expression, and strong protein production capacity, performing a pure line screening on the flax callus was necessary. The loose positive callus was made into a cell suspension and sieved to obtain small cell clusters and single cells, while pure cell clusters were obtained by the method of the nursing culture and then prepared for the establishment of suspension cell lines. This process avoided the toxicity of the toxic substances secreted by the dead cells during the antibiotic screening process when the suspension cells were directly cultured with the calluses. As shown in Fig. 4B, after 2 weeks of a nursing culturing of positive cells, the cell mass exhibited an apparent proliferation. When the diameter of the cell mass increased to about 5 mm in diameter, a GFP fluorescence-positive cell mass was detected, removed from the filter paper, and transplanted to a solid medium for direct culture. When the purified cell mass was cultured to a sufficient quantity, the quality and yield of the target protein could be detected. Qualified cell lines would construct high-quality suspension cell lines.

## Discussion

Changes in diet and lifestyle have led to a dramatic increase in the incidence of diabetes worldwide. Both type-1 and people with type-2 diabetes use insulin, and people with type-2 diabetes require large doses of insulin at a later stage (Onyechi et al. 1998; Beneyto et al. 2021; Slavin et al. 2021). The dramatic increase in the number of diabetic patients worldwide, as well as the exploration of alternative methods of insulin administration (such as inhalation or oral administration), are bound to increase the demand for recombinant insulin shortly (Beauverd et al. 2012; Nicolucci et al. 2020). Due to production capacity constraints and high production costs, current production technologies will not be able to meet the growing insulin demand. The development of insulin has gone through a hundred years of history from extracting animal insulin, chemical synthesis, and biosynthesis of recombinant insulin in recent years (Panahi et al. 2004; Nykiforuk et al. 2006; Ahmadabadi 2021). Currently, recombinant human insulin is mainly produced in *Escherichia coli* or yeast. Using the *E. coli* expression system, insulin precursors are produced as inclusion bodies; ultimately, fully functional polypeptides are obtained through solubilization and refolding procedures. Yeast-based expression systems produce soluble insulin precursors secreted into the culture’s supernatant. In addition, mammalian cells, transgenic animals, and plant expression systems have also been used as hosts for recombinant insulin production (Contreras et al. 2020; Ghasemi et al. 2021).

Plants are gradually becoming an essential recombinant protein production platform due to their low cost, ease of large-scale production, absence of risk of contamination by mammalian pathogens, and their ability to post-translationally modify expressed proteins (Trexler et al. 2002; Obembe et al. 2011; Long et al. 2022). The use of transgenic plants as a natural bioreactor for the production of recombinant proteins has recently attracted more and more attention, and a variety of medicinal proteins (that have been successfully used in clinical practice) has already been produced by transgenic plants (Buyel 2018; Lico et al. 2020).

Flax has received more and more attention in recent years and has unique advantages as a plant bioreactor. Moreover, the flax genome is relatively small, and genetic manipulation is relatively easy (Wang et al. 2012). The sequencing of the flax genome is gradually being completed, and the results of genome sequencing are more conducive to the application of genetic engineering technology in the genetic transformation of flax. In addition, Longya-10 flax seed is moderate in size, easy to operate, and has a high germination rate after surface sterilization. Hypocotyls of sterile flax seedlings were used as explants, and the callus induction from hypocotyls showed higher induction efficiency. The optimization of the induction culture system of the flax callus and the expression of recombinant protein from vigorous flax calluses have the advantages of low cost, convenient preparation, storage, and transformation. The proliferation rate of the flax callus is fast, and the proliferation amount is large, which has significant advantages for the rapid and mass production of target products. Furthermore, compared to field cultivation, this system enlarges the plant bioreactor and avoids the problem of pollen drift.

In this study, the wild-type flax hypocotyl was used as an explant to directly regenerate flax seedlings from plant organs. At the same time, the callus induced by the hypocotyl was also re-differentiated into flax seedlings. The induction of regenerated shoots on the transgenic flax hypocotyls and calluses is underway. The method of establishing a pure line of single cells and cell clusters was explored. As a result, single cells and small cell clusters were separated from the suspension cell fluid to obtain a purified positive flax cell line through the nursing culture method and then establish a flax suspension cell line. The purpose of this process was to get a pure and purified cell mass from the complex callus, including negative cells, foreign gene insertion cells at different positions in the genome, and cells that lose the target gene that is conducive to the establishment of positive plant suspension cells as well as to be used as quality control for the production of target proteins. The critical concentration of selectable marker genes is significant for screening transgenic products. Determining the critical concentration of antibiotic resistance in plant tissues is not intuitive. The hypocotyl differentiation status was observed in this study to obtain an accurate critical concentration of antibiotic resistance. Moreover, the method of regularly weighing the callus proliferation was repeated many times to get an accurate crucial concentration of the hygromycin resistance. It is expected that a simple and fast method for determining the antibiotic concentration suitable for plant tissue screening will be developed. In this study, a *fusion gene* of recombinant *insulin* and *GFP* was designed, and the *target gene* was linked to the N-terminus of the GFP. According to the direction of the peptide chain synthesis, the labeled protein can be synthesized after the target protein is synthesized. In this way, the detection of DNA and RNA can even be skipped, and it is not only intuitive and quick to verify whether the exogenous gene is successfully expressed but also helps us to monitor the time and state of the protein expression, as well as the screening of positive calluses and cells. The fusion protein expression demonstrated the feasibility of flax as an exogenous gene expression platform. Later experiments should consider designing suitable restriction sites between the target gene and the marker gene and the tag gene for protein purification. In the future, we will undertake a series of studies to (i) analyze the expression of human insulin in flax from the aspects of transcription, translation, and posttranslational modification, (ii) use circular dichroism, nuclear magnetic resonance, and other techniques to analyze the structure of the insulin expressed by flax, and (iii) examine the function of the insulin expressed by flax.

In summary, we have established a flax callus culture system suitable for insulin expression. By optimizing the conditions of the flax callus induction, transformation, screening, and verification of a transgenic callus, we have provided an effective way to obtain insulin. Future work will focus on the purification and biological activity of insulin to achieve a large-scale production of biologically active insulin. The flax callus culture system provides a feasible, cheap, and environmentally friendly platform for producing bioactive proteins.

### Electronic supplementary material

Below is the link to the electronic supplementary material.

Supplementary Material 1

Supplementary Material 2

## Data Availability

The datasets used and/or analyzed during the current study are available from the corresponding author upon reasonable request.
