# Supplementary figures and images for: Construction and optimization of a genetic transformation system for efficient expression of human insulin-GFP fusion gene in flax
Source: Bioresour Bioprocess. 2024 Aug 27;11(1):83. doi: 10.1186/s40643-024-00799-9 (PMC11349960; doi:10.1186/s40643-024-00799-9)

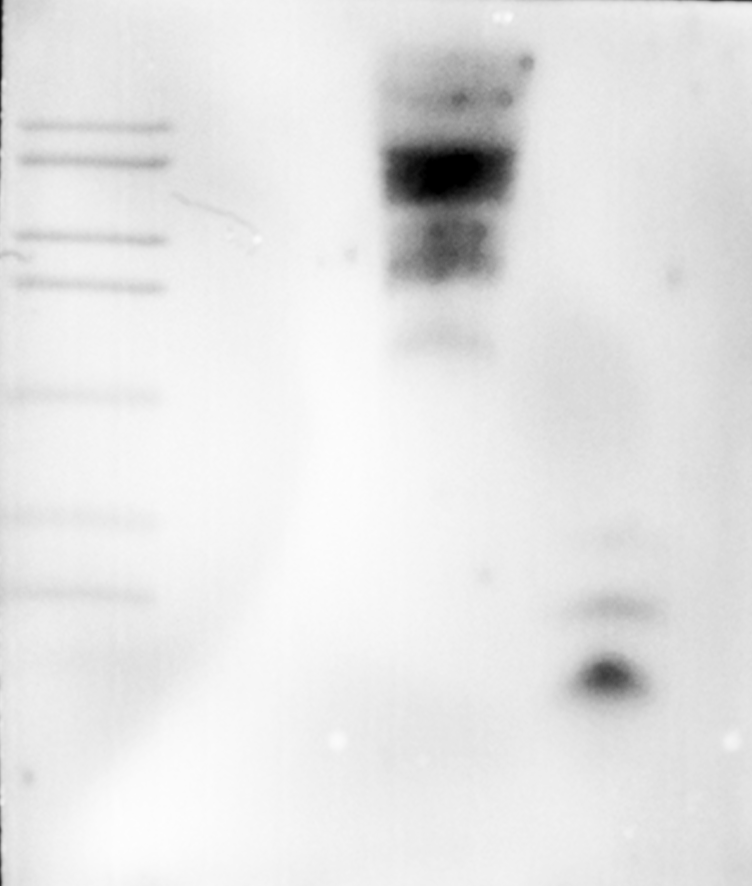

Supplement: Supplementary file 1 — Supplementary Material 1 [file 40643_2024_799_MOESM1_ESM.tif]

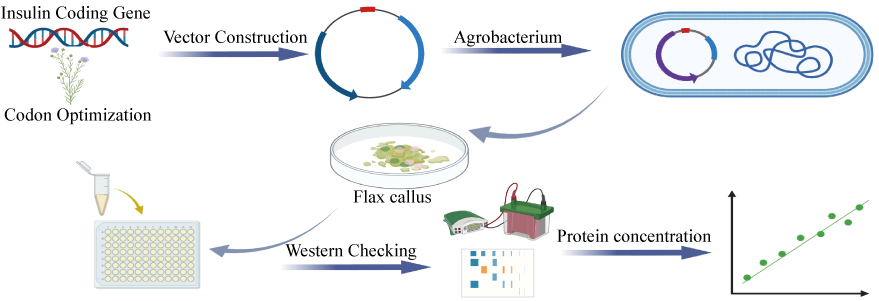

Supplement: Supplementary file 2 — Supplementary Material 2 [file 40643_2024_799_MOESM2_ESM.jpg]
